# Editorial: Exploring the interplay of interoception in emotion, cognition, and mental health

**DOI:** 10.3389/fnins.2025.1676040

**Published:** 2025-09-05

**Authors:** Daisuke Ueno, Kenta Kimura, Hideki Ohira, Jin Narumoto

**Affiliations:** ^1^Department of Psychiatry, Graduate School of Medical Science, Kyoto Prefectural University of Medicine, Kyoto, Japan; ^2^Graduate School of Contemporary Society, Kyoto Women's University, Kyoto, Japan; ^3^Graduate School of Humanities, Hosei University, Tokyo, Japan; ^4^Graduate School of Informatics, Nagoya University, Nagoya, Japan

**Keywords:** interoception, autonomic nervous system, multisensory integration, predictive processing, emotion regulation, self-representation, premenstrual syndrome, lifespan development

## 1 Introduction

### 1.1 Defining interoception

Interoception refers to the neural processing of signals originating within the body, encompassing afferent sensory information that arises from the internal organs and viscera, as well as efferent responses crucial for homeostasis. Originally conceptualized primarily as visceral sensation, contemporary perspectives now define interoception more broadly to include the sensing, interpreting, and regulating of bodily signals such as heartbeats, respiration, gastrointestinal activity, and autonomic fluctuations (Tsakiris and Critchley, 2016). More recently, definitions that include the endocrine and immune systems have also been proposed (Chen et al., 2021). Core brain regions implicated in interoceptive processing include the insular cortex, anterior cingulate cortex (ACC), and subcortical regions within the autonomic nervous system (Craig, 2002; Critchley and Harrison, 2013).

Recent literature emphasizes the multidimensionality of interoception, particularly distinguishing interoceptive accuracy—the objective ability to detect internal bodily signals—from interoceptive sensibility—subjective awareness and beliefs about internal sensations. This differentiation is supported by studies showing distinct neural correlates and behavioral outcomes associated with each dimension across clinical and nonclinical populations (Garfinkel et al., 2015; Murphy et al., 2017).

Moreover, interoceptive signals are deeply integrated with exteroceptive cues, influencing sensorimotor synchronization, bodily self-consciousness, and complex emotional experiences. Prior research demonstrates this interplay clearly, suggesting that coherent integration between internal bodily signals and external sensory stimuli underpins various perceptual and motor processes (Ainley and Tsakiris, 2013; Suzuki et al., 2013).

Collectively, contemporary views position interoception as a foundational, multidimensional construct intricately linked with cognition, emotion, and embodied self-experience.

### 1.2 Aims of this Research Topic

Given these theoretical and translational challenges, this Research Topic aimed explicitly to bridge foundational theory and real-world applicability in the domain of interoception. Contributions were invited across several specific dimensions:

1. Novel research papers examining interoception, featuring behavioral, and neuroimaging data.
2. Studies documenting how experiences manifest in life-span development of interoception or in various mental health disorders (e.g. depression, anxiety, somatic symptom disorder, or autism spectrum disorder).
3. Negative findings and failed direct or conceptual replication studies that are beyond the pilot stage, even if replication was not the primary goal, as we believe that sharing these studies will also have a positive impact on experimental planning and increase the rate of knowledge acquisition on interoception.
4. Meta-analyses or reviews that conform with the aim of the topic.

Collectively, the selected contributions exemplify these varied perspectives, strengthening the dialogue between fundamental interoceptive mechanisms and their practical implications.

## 2 Scope of this Research Topic

### 2.1 Overview of the six contributions

This Research Topic comprises six contributions covering diverse but interconnected facets of interoception.

Zhang et al. demonstrated how spatially informative rhythmic stimuli enhance sensorimotor synchronization, underlining the interaction between external sensory cues and internal bodily rhythms through β-band cortico-muscular coherence.

Suzuki and Ohira uncovered a paradoxical dissociation in women with PMS, exhibiting high interoceptive accuracy but low interoceptive awareness, combined with impaired autonomic recovery post-stress—illuminating specific interoceptive profiles relevant for clinical intervention.

Sato and Saito posited weaker subjective-facial emotional coherence as a strategic emotional regulation mechanism among older adults, framing interoceptive decoupling as potentially adaptive across the lifespan.

Cao proposed a cognitive-emotional schema model of nostalgia, arguing that interoceptive signals linked to social belongingness fundamentally shape nostalgic experiences, illustrating the complex interplay of body signals and cognitive-affective processes.

Shibata et al. showed that cardiac synchrony alone did not universally modulate self-attribution; instead, effects emerged selectively among individuals with high interoceptive accuracy, highlighting crucial boundary conditions for interoceptive influence on self-related judgments.

Kaneno et al. provided evidence that interoceptive sensibility specifically predicted body ownership in moving rubber-hand illusions, whereas interoceptive accuracy affected agency in context-dependent ways, demonstrating distinct interoceptive pathways shaping bodily self-awareness.

Together, these studies underscore the multidimensional, context-sensitive nature of interoception in shaping cognition, emotion, and bodily self-experience.

### 2.2 Why it matters

Understanding interoception is crucial due to its pervasive influence across a spectrum of psychological, cognitive, and emotional domains. Interoceptive signals form the foundation upon which subjective experiences of emotion and self-awareness are constructed (Craig, 2002; Seth, 2013; Barrett and Simmons, 2015). For instance, interoceptive dysfunction has been implicated in various mental health disorders such as anxiety, depression, somatic symptom disorders, and eating disorders (Khalsa et al., 2018; Paulus and Stein, 2010). This Research Topic's contributions underscore such clinical significance: Suzuki and Ohira's work connects disrupted interoceptive awareness and autonomic regulation to emotional vulnerability in PMS, suggesting potential avenues for targeted biofeedback interventions.

Interoception also contributes critically to adaptive coping and emotion-regulation strategies across the lifespan. For example, alterations in interoceptive processing have been associated with aging-related changes in emotional coherence and well-being (Mendes, 2010; Shiota and Levenson, 2009). Sato and Saito posit reduced coherence between subjective emotional experience and facial expressions as a potentially adaptive coping mechanism in older adults, implying interoception's involvement in regulating emotional expression and promoting wellbeing in aging. Additionally, Cao theorizes that nostalgic experiences, guided by interoceptively-mediated social needs, operate as cognitive-emotional schemas that restore psychological continuity and meaning—highlighting the interplay between bodily sensations and higher-order cognition.

Moreover, the ability of interoceptive signals to dynamically shape the boundaries of the self has profound implications for understanding embodiment and agency. Prior experimental studies have demonstrated how cardiac and proprioceptive signals modulate self-attribution, agency, and body ownership, thereby reinforcing the centrality of interoception in maintaining coherent self-experiences (Aspell et al., 2013; Tsakiris, 2017). Shibata et al. and Kaneno et al. show experimentally how cardiac and proprioceptive signals influence self-attribution and body ownership, illustrating the crucial role interoception plays in maintaining coherent self-representation.

Given its broad-ranging impact on mental health, emotion regulation, self-awareness, and cognitive functioning, interoception emerges as not merely a bodily sense, but as a cornerstone of human subjective experience, opening novel avenues for both basic and translational research.

### 2.3 Gaps between theory and application

Although significant progress has been made in elucidating the mechanisms underlying interoception, notable gaps persist between theoretical frameworks and real-world applications. Firstly, existing research often treats interoceptive dimensions—such as accuracy, awareness, and sensibility—as interchangeable constructs. However, the evidence provided by Suzuki and Ohira and Kaneno et al. clearly indicates distinct outcomes when these dimensions diverge, emphasizing the necessity to refine and distinguish interoceptive measurements for clinical and applied contexts.

Secondly, despite the widespread use of predictive-coding theories to conceptualize interoception (Seth, 2013), direct empirical demonstrations remain limited primarily to basic sensorimotor contexts, as shown by Zhang et al.. Translating these theories into complex emotional or cognitive phenomena such as nostalgia (Cao) or age-related emotional coping (Sato and Saito) is still challenging.

Thirdly, a translational bottleneck persists. Many laboratory paradigms, such as the rubber-hand illusion (Kaneno et al.) and heartbeat-synchronization tasks (Shibata et al.), provide robust mechanistic insights but often lack ecological validity, limiting generalization to everyday experiences. Additionally, methodological inconsistencies, especially with popular tasks like heartbeat counting, pose reliability concerns across studies.

Finally, clear and validated intervention protocols remain scarce despite identifying promising clinical targets, such as autonomic dysregulation in PMS or multisensory sensorimotor integration in motor disorders. Addressing these gaps is critical for advancing the translational potential of interoception research.

## 3 Synthesis and future directions

### 3.1 Converging themes

Three core themes emerged clearly across these contributions: (1) multisensory integration as a fundamental pathway through which interoception modulates perception-action coupling and self-representation; (2) the critical differentiation between objective (accuracy) and subjective (awareness, sensibility) dimensions of interoception; and (3) distinct adaptive and maladaptive emotion-regulation patterns mediated by interoception throughout the lifespan.

### 3.2 Methodological advances and caveats

These papers collectively represent a diverse methodological toolbox—ranging from EEG and EMG coherence analyses to psychophysiological stress paradigms, bodily illusions, and theoretical syntheses. However, methodological diversity also highlights important caveats, including small or homogeneous samples, limited ecological validity, and measurement reliability challenges. Future research should address these issues via preregistration, data-sharing, and methodological standardization.

### 3.3 Translational opportunities

Several promising translational pathways emerge clearly from the featured contributions:

Clinical interventions targeting interoceptive awareness could be designed to address autonomic dysregulation in conditions like premenstrual syndrome (PMS), as suggested by findings on impaired autonomic recovery after stress.

Neuro-rehabilitation protocols employing multisensory rhythmic stimuli could leverage insights into sensorimotor synchronization, as demonstrated through enhanced β-band cortico-muscular coherence.

Emotion-regulation training informed by interoceptive mechanisms might support adaptive emotional coping in older populations, reflecting strategic reductions in subjective-facial emotional coherence.

Digital and virtual reality (VR) therapies could use cardiac synchronization to facilitate improved self-attribution and body ownership, thus enhancing embodied interventions in clinical populations.

### 3.4 Future research agenda

To capitalize on the translational potential demonstrated in these studies, future research should prioritize:

Longitudinal studies examining how interoceptive accuracy and sensibility evolve across the lifespan, providing insights into developmental trajectories of emotional coping mechanisms and sensorimotor integration.

Large-scale, multimodal studies integrating psychophysiological measures, neuroimaging, and ecological momentary assessments to clarify relationships between interoceptive signals and complex emotional states such as nostalgia.

Computational modeling to further refine predictive-coding approaches, particularly in explaining how interoceptive processes underpin multisensory integration and bodily self-awareness.

Cross-cultural and gender-specific studies to explore variations in interoceptive mechanisms and their clinical implications, for instance, identifying unique interoceptive profiles in women experiencing PMS.

Robust randomized controlled trials systematically evaluating interoceptive training methods, such as biofeedback and rhythmic sensory entrainment, for enhancing interoceptive accuracy, sensibility, and subsequent mental health outcomes.

## 4 Conclusion

Collectively, the studies presented in this Research Topic significantly advance our understanding of interoception's intricate connections with emotion, cognition, self-awareness, and mental health. By bridging fundamental theory and real-world implications, these contributions provide critical insights into how internal bodily signals shape human subjective experience. Future interdisciplinary collaborations inspired by these findings hold the potential to unlock innovative, personalized interventions capable of enhancing psychological and physical wellbeing across diverse populations and lifespan contexts.
